# Placenta-specific *Slc38a2*/SNAT2 knockdown causes fetal growth restriction in mice

**DOI:** 10.1042/CS20210575

**Published:** 2021-08-31

**Authors:** Owen R. Vaughan, Katarzyna Maksym, Elena Silva, Kenneth Barentsen, Russel V. Anthony, Thomas L. Brown, Sara L. Hillman, Rebecca Spencer, Anna L. David, Fredrick J. Rosario, Theresa L. Powell, Thomas Jansson

**Affiliations:** 1 Department of Obstetrics and Gynecology, University of Colorado Anschutz Medical Campus, Aurora, CO 80045, U.S.A.; 2 Department of Maternal and Fetal Medicine, Elizabeth Garrett Anderson Institute for Women’s Health, University College London WC1E 6HX, U.K.; 3 Department of Biomedical Sciences, Animal Reproduction and Biotechnology Laboratory, Colorado State University, Fort Collins, CO 80523, U.S.A.; 4 Department of Neuroscience, Cell Biology and Physiology, Wright State University Boonshoft School of Medicine, Dayton, OH 45435, U.S.A.; 5 Leeds Institute of Cardiovascular and Metabolic Medicine, University of Leeds, Leeds LS2 9NL, U.K.; 6 Department of Pediatrics, University of Colorado Anschutz Medical Campus, Aurora, CO 80045, U.S.A.

**Keywords:** amino acid transport system A, fetal growth restriction, lentivirus, maternal-fetal exchange, MeAIB, syncytiotrophoblast

## Abstract

Fetal growth restriction (FGR) is a complication of pregnancy that reduces birth weight, markedly increases infant mortality and morbidity and is associated with later-life cardiometabolic disease. No specific treatment is available for FGR. Placentas of human FGR infants have low abundance of sodium-coupled neutral amino acid transporter 2 (*Slc38a2*/SNAT2), which supplies the fetus with amino acids required for growth. We determined the mechanistic role of placental Slc38a2/SNAT2 deficiency in the development of restricted fetal growth, hypothesizing that placenta-specific *Slc38a2* knockdown causes FGR in mice. Using lentiviral transduction of blastocysts with a small hairpin RNA (shRNA), we achieved 59% knockdown of placental *Slc38a2*, without altering fetal *Slc38a2* expression. Placenta-specific *Slc38a2* knockdown reduced near-term fetal and placental weight, fetal viability, trophoblast plasma membrane (TPM) SNAT2 protein abundance, and both absolute and weight-specific placental uptake of the amino acid transport System A tracer, ^14^C-methylaminoisobutyric acid (MeAIB). We also measured human placental *SLC38A2* gene expression in a well-defined term clinical cohort and found that *SLC38A2* expression was decreased in late-onset, but not early-onset FGR, compared with appropriate for gestational age (AGA) control placentas. The results demonstrate that low placental *Slc38a2*/SNAT2 causes FGR and could be a target for clinical therapies for late-onset FGR.

## Introduction

Fetal growth restriction (FGR) is a pregnancy complication characterized by abnormal placental function and low birth weight [1]. FGR severely compromises neonatal survival [2] and infant lifelong health [3,4]. There is currently no cure and the underlying mechanisms remain poorly understood. FGR is associated with reduced placental amino acid transport capacity and reduced umbilical concentrations of amino acids [5–8], which provide substrate for fetal tissue accretion [9–11] and stimulate the secretion of growth hormones *in utero* [12,13]. In particular, System A amino acid transport capacity is low in the maternal-facing syncytiotrophoblast microvillous plasma membrane of human FGR placentas [14–16], suggesting that impaired placental System A transport contributes to the deficit in fetal nutrient supply.

System A transporters mediate sodium-dependent, active accumulation of small neutral amino acids from the maternal blood into the syncytiotrophoblast epithelium (reviewed in [17]). Active transport of amino acids into the placenta is critical for their transepithelial transfer to the fetus, where amino acid concentrations are higher than in the mother [6,7,18]. Amino acids accumulated by System A also drive the placental uptake of large, branched-chain amino acids, including essential amino acids like leucine, via microvillous membrane System L exchangers [19–21]. System A activity is attributed to three sodium-coupled neutral amino acid transporter (SNAT) protein isoforms: SNAT1, SNAT2 and SNAT4, which have all been localized to the human placental microvillous membrane [22–25]. Human FGR has been linked to reduced placental SNAT2 abundance [22,26].

Experimental manipulations that specifically reduce plasma membrane SNAT2 abundance in primary human trophoblast cells inhibit System A activity *in vitro* [27], and impaired placental System A activity temporally precedes FGR in undernourished rats and baboons [28–30], suggesting that SNAT2 deficiency mechanistically contributes to the development of FGR. In pregnant rodents, systemic pharmacological inhibition of System A activity, using a non-metabolizable, competitive synthetic substrate, reduces fetal weight [31]. Moreover, in mouse embryos, placental and fetal size are both diminished by global deletion of either *Slc38a1, Slc38a2* or *Slc38a4*, which encode SNAT1, SNAT2 and SNAT4, respectively [32]. Global deletion of *Slc38a2* also reduces perinatal survival in mouse pups [33]. Other genetic, environmental and hormonal manipulations that reduce intrauterine growth in mice frequently tend to decrease placental SNAT2 abundance and System A activity, measured by unidirectional maternal–fetal clearance of the tracer methylaminoisobutyric acid (MeAIB) [34–38]. However, to date, there has been no demonstration of a cause-and-effect relationship between low placental SNAT2 abundance and reduced fetal growth *in vivo*.

FGR is also strongly associated with lower fetal insulin, glucose-stimulated insulin secretion (GSIS), islet size and β-cell number [39]. Decreased amino acid transfer from the placenta to the fetus is concomitant with defective fetal pancreatic islet structure and function in FGR sheep fetuses while chronic fetal amino acid infusion restores fetal GSIS, pancreatic vascularity, islet size and β-cell mass [40,41]. However, there is no definitive *in vivo* genetic evidence of placental amino acid transporter involvement in regulating fetal islet function.

Clinically, FGR is increasingly recognized as either early- or late-onset disease. By definition, early- and late-onset FGR are diagnosed before and after 32 weeks of gestation, respectively, and have distinct phenotypes [42–44]. Early-onset FGR is strongly associated with pre-eclampsia, maternal vascular malperfusion of the placenta, abnormal umbilical artery Doppler measurements, fetal hypoxia, preterm delivery and fetal demise [44–46]. Contrastingly, late-onset FGR is less commonly associated with pre-eclampsia, vascular abnormalities or fetal mortality but affects a larger proportion of pregnancies, is a common cause of stillbirth and carries a similar burden of poor long-term neurodevelopmental and cardiometabolic outcomes in the neonate [44]. The respective contribution of placental amino acid transport to early- and late-onset FGR has not been investigated, and the etiology of late-onset FGR is particularly unclear.

We hypothesized that placenta-specific *Slc38a2* knockdown causes FGR and impairs fetal islet GSIS in mice. We used a lentiviral vector to deliver a small hairpin (sh) RNA (shRNA) gene silencing construct to the blastocyst trophectoderm in early gestation [47–51] and subsequently determined placental and fetal weight, *in vivo* placental amino acid transport capacity and placental amino acid transporter abundance near term. We also measured GSIS in isolated fetal islets from fetuses with placenta-specific *Slc38a2* knockdown. Finally, we quantified human placental *SLC38A2* gene expression in placentas from pregnancies with early- or late-onset FGR, compared with appropriate for gestational age (AGA) control placentas.

## Materials and methods

### Lentiviral vectors

The shRNA gene silencing plasmid, targeting the mouse *Slc38a2* mRNA (Slc38a2KD), was based upon the pLKO.1 vector [52] and obtained from a commercial vendor (VectorBuilder Inc., Chicago, IL, U.S.A.). Targeting sequence (TCACGGTCCCAGTAGTTATTT), hairpin loop (CTCGAG) and guide sequence (AAATAACTACTGGGACCGTGA) were inserted in series into the plasmid backbone, downstream of the human U6 small nuclear 1 promoter. The plasmid backbone also contained an enhanced green fluorescent protein (EGFP) open reading frame expressed from the human phosphoglycerate kinase 1 promoter and an ampicillin resistance gene. A control plasmid (SCR), with scrambled targeting sequence (CCTAAGGTTAAGTCGCCCTCG), was constructed similarly. *Escherichia coli* glycerol stocks of both plasmids were amplified in overnight cultures of LB broth with 100 μg.ml^−1^ ampicillin, then DNA was isolated using a commercially available kit (Plasmid Maxi kit, Qiagen). Targeting and control plasmids were then packaged into vesicular stomatitis virus G pseudotyped lentiviral particles by co-transfecting 293FT cells with the isolated DNA (440 ng.ml^−1^, with Qiagen Polyfect 1% v/v in culture medium), together with packaging (330 ng.ml^−1^ pCMV delta 8.9) and envelope plasmids (140 ng.ml^−1^ pCMV-VSV-G [53]), obtained from the Functional Genomics Shared Resource at the University of Colorado Cancer Centre. Human 293FT (female embryonic kidney epithelial) cells were purchased from Thermo Fisher Scientific and cultured at 37°C in complete medium (Dulbecco’s modified Eagle’s medium with 10% fetal bovine serum, 0.1 mM non-essential amino acids, 6 mM glutamine, 1 mM sodium pyruvate and 1% pen–strep). Lentiviral particles were isolated from the filtered culture medium 72 h after transfection by ultracentrifugation over 20% sucrose (22000 rpm, 2 h) then resuspended in PBS. The functional titer of each batch of lentivirus, in transforming units per ml, was quantified by transducing 293FT cells with serial dilutions of the viral suspension then determining the percentage of EGFP fluorescent cells using flow cytometry.

### Lentiviral transduction of mouse blastocysts and surgical embryo transfer

All animal procedures were carried out at the University of Colorado with approval from the Institutional Animal Care and Use Committee (Protocol #344). B6D2F1 mice were purchased from Charles River Laboratories (Wilmington, MA, U.S.A.) and CD-1 mice were purchased from Jackson Laboratories (Bar Harbor, ME, U.S.A.). All animals were maintained under standard 14-h:10-h light:dark conditions with *ad libitum* access to food and water. Female B6D2F1 mice (*n*=140), aged <4 weeks, were injected with pregnant mare serum gonadotrophin (5 I.U., i.p., Prospec, East Brunswick, NJ, U.S.A.) and human chorionic gonadotrophin (5 I.U., i.p., Sigma–Aldrich, St Louis, MO, U.S.A.) to induce superovulation, then mated overnight with stud B6D2F1 males. Successfully mated females were identified by the presence of a copulatory plug the following morning, which was designated embryonic day (E) 0.5 (term ≈ E19.5). On E3.5, pregnant females were killed by CO_2_ asphyxiation and cervical dislocation, their uteri excised and each horn flushed with 5 ml of pre-warmed M2 medium (M7167, Sigma–Aldrich, St. Louis, MO, U.S.A.). Flushed blastocysts were denuded of the zona pellucida by serially incubating in three drops of acidic Tyrodes solution (∼10 s each), then washed and incubated in embryo culture medium (EmbryoMax Advanced KSOM Embryo medium, MR-101-D, Millipore). The trophectoderm was then transduced with either Slc38a2KD or SCR by incubating batches of five to ten blastocysts with either 5 × 10^5^ or 5 × 10^6^ transforming units of lentivirus in a total volume of 20 µl, for 4 h. Blastocysts were subsequently washed in 12 drops of embryo culture medium and surgically transferred to pseudopregnant female CD-1 recipients. All *in vitro* manipulations were performed at 37°C and under low-light conditions.

Female CD-1 embryo recipient mice (*n*=74) were mated overnight with vasectomized B6D2F1 males, to induce pseudopregnancy, and underwent surgical embryo transfer 2.5 days post-copulation. Recipients were given pre-operative analgesia (meloxicam, 1 mg/kg, i.p.) then anesthetized with isofluorane (2%, inhaled). With the animal in sternal recumbency and under aseptic conditions, a 0.5-cm incision was made in the skin of the flank overlying the right ovary, 1 cm caudal to the rib cage. Ovary, oviduct and distal uterine horn were exteriorized through the body wall and a 26-ga needle used to puncture the uterus below the oviductal junction. Lentivirus-transduced blastocysts [7–12] were then gently inserted into the uterine horn in a minimal volume of culture medium. The uterus and oviducts were then returned to the body cavity, the body wall closed with a single absorbable suture (5.0 vicryl) and the skin closed with a 9-mm wound clip. The procedure was then repeated for the left ovary, with Slc38a2KD and SCR transduced blastocysts transferred to contralateral horns of the same uterus and randomized to left and right in each recipient. Post-operatively, recipient females were recovered from anesthesia on a heated mat and housed in pairs.

### *In vivo* transplacental amino acid clearance and tissue collection

Fetal and placental phenotype were assessed on E17.5 (*n*=16) or E18.5 (*n*=58). System A and System L-mediated placental amino acid transport capacity were measured in a subset of E18.5 pregnant recipient females (*n*=16), by measuring maternal–placental and maternal–fetal clearance of non-metabolizable, radiolabeled ^14^C-methylaminoisobutryic acid (^14^C-MeAIB) and ^3^H-leucine, respectively. Dams were anesthetized using ketamine (60 mg/kg) and xylazine (6 mg/kg, both *i.p.*) and placed on a heated mat. The lateral tail vein was cannulated and flushed with heparinized saline using a 28-ga needle attached to a 0.5-ml insulin syringe, via polyethylene (PE20) tubing (0.38 mm I.D., ∼12 cm length, B.D. Intramedic 427405, Becton Dickinson, NJ, U.S.A.). A combined bolus of ^14^C-MeAIB (50 µCi/kg, specific activity 58.7 mCi/mmol) and ^3^H-leucine (250 µCi/kg, specific activity 60000 mCi/mmol) was delivered to the tail vein, with flushing. At exactly 1.5, 3 or 4.5 min later, the dam was killed with sodium pentobarbital (390 mg/ml, 100 µl, i.v.) and a cardiac blood sample was rapidly collected into a heparinized syringe. The blood was then centrifuged (12000 rpm, 4 min, 4°C) and the supernatant plasma separated and frozen. The uterus was exposed via laparotomy and the number of viable fetuses and non-viable resorptions counted in each horn. Fetuses and placentas were dissected from the uterus and membranes, blotted briefly and weighed. Individual fetuses and placentas were then solubilized in Biosol (1 ml for placenta, 3 ml for fetus, National Diagnostics) at 55°C, overnight. Aliquots of maternal plasma and fetal digestate were subsequently cleared with hydrogen peroxide and their radioactivity determined by liquid scintillation counting. Initial experiments determined that the ratio of fetal to maternal plasma radioactivity per unit volume increased linearly with respect to time until at least 3 min after injection of both tracers, indicating unidirectional maternal–fetal flux in this time period (Supplementary Figure S5). Therefore, a clearance curve of maternal plasma radioactivity per ml versus time from tracer injection was constructed from these experiments and used to calculate net maternal–placental and maternal–fetal clearance of ^14^C-MeAIB and ^3^H-leucine for all pregnant dams killed at 1.5 or 3 min after tracer injection, as described [54]. The remaining recipient dams that did not undergo tracer measurements (*n*=58) were killed by CO_2_ asphyxiation and cervical dislocation and fetuses and placentas counted, weighed and snap-frozen in liquid N_2_. In a subset of litters (*n*=6), fetal liver, heart, brain and skeletal muscle were dissected and pooled for SCR and Slc38a2KD fetuses, within each litter. In a different subset of litters (*n*=3), placentas were dissected into their constituent labyrinthine and junctional zones and frozen for gene expression analysis. In all cases, embryo implantation rate was determined as the sum of viable and resorbed conceptuses divided by number of embryos transferred, while viability rate was determined as the number of viable conceptuses divided by number of embryos transferred.

### Gene expression analysis

RNA was extracted from frozen placentas and fetal tissues and reverse transcribed using commercially available kits (RNeasy Plus Mini kit, Qiagen and High-Capacity cDNA RT kit, Invitrogen). The expression of *Slc38a1*, *Slc38a2* and *Slc38a4* was determined by SYBR Green qRT-PCR using the relative standard curve method, relative to RNA28S. Primer sequences are given in Supplementary Table S4.

### Trophoblast plasma membrane isolation

For analyses of transporter and signaling protein abundance, pools of SCR and Slc38a2KD placentas were created from multiple litters. Pools contained between 3 and 11 individual placentas and were matched such that each Slc38a2KD pool (*n*=10) comprised placentas from the same litters as the corresponding SCR pool (*n*=10). Pooled frozen tissue was homogenized in buffer D (250 mM sucrose, 10 mM Hepes-Tris and 1 mM EDTA (pH 7.4)) with protease and phosphatase inhibitors. Trophoblast plasma membrane (TPM) vesicles were isolated from the homogenate using differential ultracentrifugation and Mg^2+^ precipitation, as previously described [55]. Briefly, homogenates were serially centrifuged at 10000×***g*** (10 min, 4°C) and 125000 rpm (30 min, 4°C) to remove tissue debris and nuclei. The resultant pellet was resuspended and the TPM precipitated by addition of MgCl_2_ (12 mM) with stirring, on ice. Precipitated TPM was then isolated by further ultracentrifugation (33000 rpm, 30 min, 4°C), resuspended and vesiculated using a Dounce homogenizer. Homogenate and TPM protein content were determined by bicinchoninic acid assay and the enrichment of the preparation determined by the ratio of alkaline phosphatase activity, per unit protein in the TPM compared with crude homogenate. Average TPM enrichment ratios were similar in SCR (8.9 ± 1.2) and Slc38a2KD (7.7 ± 0.8) pools (*P*=0.275).

### Fetal pancreatic islet isolation and measurement of glucose‐stimulated insulin secretion

Fetal islet function was measured in a subset of litters at E18.5 (*n*=7). Fetuses were dissected, glucose concentrations in trunk blood measured using a hand-held glucometer and fetal pancreata dissected and pooled for SCR and Slc38a2KD conceptuses within each litter. Pancreata were digested at 37°C for 10–15 min with Hanks’ balanced salt solution (Life Technologies) containing 2.5% bovine serum albumin (BSA) (wt/vol; Sigma), 0.35 g/l NaHCO_3_ (Sigma) and 2 mg/ml Collagenase P (Roche). Digested tissue was then washed in the cold, supplemented Hanks’ balanced salt solution without collagenase. Islets were isolated by histopaque gradient centrifugation and washed. Isolated islets were snap-frozen in liquid N_2_ or cultured (50/sample) in RPMI medium for 2 h, preconditioned in Krebs–Ringer bicarbonate buffer for 90 min and then incubated for 60 min with either 2.8 or 10.0 mM glucose in the same buffer. Subsequently, the supernatant was collected and stored at −80°C for later analysis of insulin content by ELISA (Alpco, NH, U.S.A.).

### Immunoblotting

SNAT2 abundance in TPM (*n*=9 paired SCR and Slc38a2KD pools) and Pdx1 (pancreatic and duodenal homobox 1) abundance in fetal islet lysates (*n*=6 paired SCR and Slc38a2KD pools) were determined by Western blot. Equal amounts of protein from each sample pool were resolved on a polyacrylamide gel, under reducing, denaturing conditions, then transferred to polyvinylidene fluoride membrane, overnight. Membranes were probed with rabbit polyclonal antibodies raised against SNAT2 [56] or a commercially available monoclonal Pdx1 antibody (#5679, Cell Signaling Technology) and visualized using an HRP-conjugated secondary antibody and enhanced chemiluminescence reaction, in an automated gel imaging system. Band intensities were quantified by densitometry and normalized for total protein load, determined by amido black staining. Amino acid response and mechanistic target of rapamycin (mTOR) signaling pathway activity in crude placental homogenates was also determined by Western blot for total and phosphorylated forms of eIF2α (Ser^51^), S6 (Ser^235/236^), 4EBP1 (Thr^37/46^) and Akt (Ser^473^).

### Analysis of human placental *SLC38A2* expression in FGR and AGA pregnancies

Placentas were collected from pregnant women who were prospectively recruited at University College London Hospital, London, U.K. All women had their Estimated Date of Delivery (EDD) calculated from their last menstrual period (LMP) confirmed by ultrasound in the first trimester. Women with pregnancies affected by early-onset FGR were defined by ultrasound-assessed estimated fetal weight (EFW) < 600 g and <3^rd^ centile, between 20 and 26 + 6 weeks of gestation [46,57]. Women with pregnancies affected by late-onset FGR were defined as having a normal sized fetus at their mid-gestation anomaly scan (EFW ≥ 10^th^ centile for gestation) but had an EFW < 10^th^ centile diagnosed after 32 weeks, and delivered at term. Women with early FGR delivered either preterm (<37 weeks of gestation) or at term (≥37 weeks of gestation). Women with AGA fetuses (10^th^–95^th^ centile) delivered at term and were recruited as part of a case–control study [58–60]. Exclusion criteria included multiple pregnancy, maternal age < 18 years, fetal structural or chromosomal abnormalities, premature preterm rupture of membranes or maternal bacterial or viral infection.

At delivery of liveborn infants, placental villous tissue was sampled from two points midway between the umbilical cord insertion and margin, dissected free of decidua and chorionic plate and snap-frozen in liquid nitrogen, or placed in RNAlater. RNA was subsequently extracted from tissue stored at −80°C, using a commercially available kit (RNeasy Fibrous Tissue Mini Kit, Qiagen). Following reverse transcription, placental *SLC38A2* expression was quantified using Taqman RT-qPCR, normalized to the geometric mean of *18S* (*ribosomal 18S RNA*), *GAPDH* (*glyceraldehyde-3-phosphate dehydrogenase*), *YWHAZ* (*tyrosine 3-monooxygenase/tryptophan 5-monooxygenase activation protein ζ*) and *B2M* (*β-2-microglobulin*).

### Statistics

Results from experimental analyses in mice are mean ± SEM unless indicated otherwise. Categorical data on implantation and viability rate were analyzed by Fisher’s exact test. Discontinuous data on litter size were analyzed by non-parametric Mann–Whitney test. Gene expression, morphometric and placental transport data were determined for every viable conceptus within each litter, then a litter mean value for SCR and Slc38a2KD conceptuses was calculated and used for statistical analysis. SCR and Slc38a2KD litter means were compared by two-tailed Student’s *t* test, paired within each litter. The linear relationship of individual fetal weights with placental *Slc38a2* expression or amino acid transport was determined by Pearson’s product–moment correlation. Transporter and signaling protein abundances determined for matched pools of SCR and Slc38a2KD placentas were also compared by paired Student’s *t* test.

Results from human subjects are median (interquartile range) for continuous data, or number (% of group total) for categorical data. Analyses were based on comparison of subjects within term-delivered AGA, late- and early-onset FGR, and preterm-delivered early-onset FGR study groups. For categorical data, proportions of each group total were compared across study groups by chi-squared test. For continuous data, normality of residuals was assessed by Shapiro–Wilk test then intergroup comparisons were made by one-way ANOVA with Sidak’s multiple comparisons post-hoc or by Kruskal–Wallis test with Dunn’s multiple comparisons post-hoc, as appropriate. Post-hoc comparisons were conducted in a planned manner, between term AGA, late- and early-onset FGR subjects, then separately between term and preterm early-onset FGR subjects. Correlation and regression analyses were performed for subjects delivering at term only. Interdependence of continuous variables was assessed by Pearson’s correlation. The effect of FGR status on placental *SLC38A2* expression, adjusted for potential confounding continuous and categorical variables, was determined using main effects multiple linear regression, with *SLC38A2* as the dependent variable. In all cases, significance was at *P*<0.05. Statistical details of experiments can be found in figure and table legends.

### Study approval

Human subjects were recruited with written informed consent and ethical approval from U.K. National Health Service Research Ethics Committees (London: Hampstead Research Ethics Committee, REC reference 15/LO/1488 and Stanmore Research Ethics Committee, REC reference 13/LO/1254).

## Results

### Placenta-specific lentiviral transduction of mouse embryos

B6D2F1 mouse blastocysts were transduced with a lentiviral shRNA vector targeting *Slc38a2* (Slc38a2KD), on day (E) 3.5 post-conception, to silence the *Slc38a2* mRNA specifically in the trophectoderm cells, which form the definitive placenta [61] (see ‘Materials and methods’ section). Control blastocysts were transduced with a scrambled, non-targeting vector (SCR). Both lentiviral vectors expressed a green fluorescent protein (GFP) reporter driven by the human phosphoglycerate kinase 1 promoter. Irrespective of shRNA construct, blastocysts transduced with either 5 × 10^5^ or 5 × 10^6^ transforming units of lentivirus, but not with lower viral titers, expressed GFP immediately after the 4-h transduction period and 24 h later (Supplementary Figure S1A). When blastocysts were surgically transferred to pseudopregnant CD-1 recipient female mice, those embryos transduced with 5 × 10^6^ transforming units produced fewer viable fetuses near term (1%, 2 fetuses/159 embryos transferred, 10 recipients) and fewer total implantations (8%, 12 implantation sites/159 embryos transferred) than embryos transduced with 5 × 10^5^ transforming units (27% viability rate, 336 fetuses/1255 embryos and 33% implantation rate, 410 implantation sites/1255 embryos; 64 recipients *P*<0.001 Fisher’s exact test, E17.5 and E18.5 combined). Therefore, subsequent analyses of intrauterine growth used blastocysts incubated with 5 × 10^5^ transforming units of lentivirus. SCR and Slc38a2KD transduced blastocysts were transferred to contralateral horns of each recipient uterus. Ninety-five percent of viable conceptuses (329/336) exhibited placenta-specific GFP reporter expression at term (Supplememtary Figure S1B) and GFP negative conceptuses (5%, 17/336) were excluded from morphometric analyses.

### Placenta-specific *Slc38a2* knockdown reduces fetal and placental weight

On E17.5, implantation and viability rate were similar in SCR and Slc83a2KD conceptuses (Supplementary Table S1). *Slc38a2* expression was 26% lower in Slc38a2KD than SCR placentas (Supplementary Figure S2A). Placenta weight (−15%), but not fetal weight, was lower in Slc38a2KD conceptuses than SCR conceptuses (Supplementary Figure S2B,C). However, there was a trend for fetal weight to be lower by 7% (*P*=0.059). We therefore sought to determine the effect of placental Slc38a2 knockdown closer to term, at E18.5, because the mouse fetus grows rapidly over this period, gaining weight by two- to three-fold between E16.5 and E18.5 [62].

**Figure 1:**
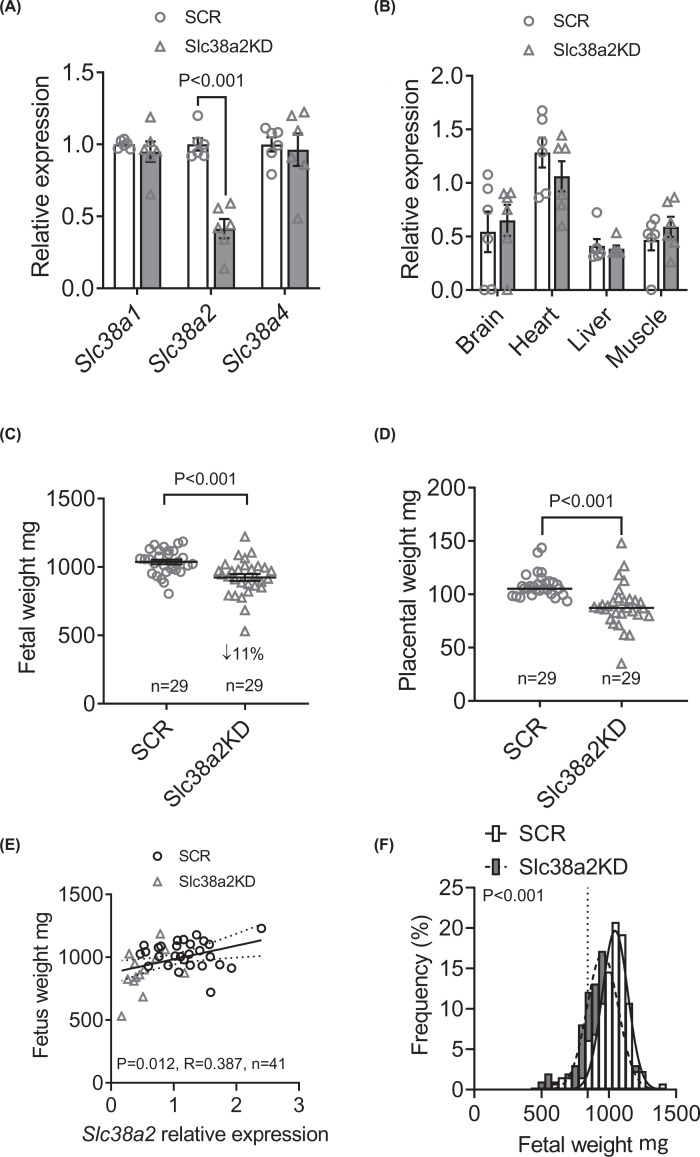
Placenta-specific Slc38a2 knockdown reduces placental and fetal weights at E18.5 (**A**) Gene expression of *Slc38a1, Slc38a2* and *Slc38a4* in placentas of SCR and Slc38a2KD conceptuses, *n*=6 litters (28 SCR placentas, 13 Slc38a2KD placentas). (**B**) *Slc38a2* expression in selected fetal tissues from SCR and Slc38a2 conceptuses, pooled from *n*=6 litters (27 SCR fetuses, 15 Slc38a2KD fetuses). (**C**) Fetal weight and (**D**) placental weight in SCR and Slc38a2KD conceptuses from *n*=29 litters (130 SCR conceptuses and 99 Slc38a2KD conceptuses). Points represent litter means. (A–D) Litter mean values for SCR and Slc38a2 compared by paired Student’s *t* test. *P*-values for statistically significant differences (*P*<0.05) given in figure. Mean ± SEM. (**E**) Correlation of fetal weight with placental *Slc38a2* expression for individual SCR (*n*=28) and Slc38a2KD (*n*=13) conceptuses. Points represent individual conceptuses. Pearson correlation coefficient (R) and *P*-value given in figure. Linear regression line with 95% confidence intervals shown. (**F**) Frequency distribution of individual fetal weights in SCR (*n*=130) and Slc38a2KD conceptuses (*n*=99). Gaussian curves fitted by least-squares non-linear regression and compared by extra sum-of-squares F-test (*P-*value given in figure). Best-fit values for curves: SCR amplitude 18.7 ± 1.2%, mean 1034 ± 7 mg, SD 101 ± 7 mg; Slc38a2KD amplitude 17.2 ± 1.3%, mean 952 ± 10 mg, SD 113 ± 10 mg. Dotted vertical line indicates 10^th^ percentile of SCR fetal weights.

On E18.5, implantation rate was similar in SCR and Slc38a2KD embryos (Table 1). However, a smaller proportion of the Slc38a2KD embryos gave rise to viable fetuses, resulting in fewer viable Slc38a2KD conceptuses than SCR conceptuses per uterine horn (Table 1). Only litters of at least four viable fetuses in total, and at least one viable fetus transduced with each virus, were used for subsequent analyses (median number of fetuses per horn in this subset: SCR, 4; Slc38a2KD, 3, *P*=0.009, *n*=29 litters). Placental *Slc38a2* mRNA expression was 59% lower in Slc38a2KD conceptuses than SCR conceptuses, whereas *Slc38a1* and *Slc38a2* expression were similar in the two groups, as expected (Figure 1A). Similarly, when placentas were dissected to separate the nutrient-transporting labyrinthine zone from the hormone-producing junctional zone, Slc38a2KD reduced *Slc38a2* mRNA expression in both zones, without altering *Slc38a1* or *Slc38a2* expression, even though basal expression of all three transporters was higher in the labyrinth than in the junctional zone (Supplementary Figure S3). *Slc38a2* mRNA expression in non-placental tissues did not differ between SCR and Slc38a2KD conceptuses, in either the fetal liver, heart, brain or skeletal muscle (Figure 1B), consistent with trophoblast-specific knockdown of the target gene.

**Table 1:** Implantation and viability rates for SCR and Slc38a2KD transduced embryos at E18.5

	SCR	Scl38a2KD	*P*-value
**Total embryos transferred (*n*)**	550	538	
**Implanted (*n*)**	195	165	
**%**	35%	31%	0.094^1^
**Viable fetus at E18.5**	178	113	
**%**	32%	21%	<0.001^1^
**Number of fetuses per uterine horn (median, min-max)**	4 (0–9)	2 (0–12)	<0.001^2^

Embryos were transferred to 58 recipient dams, of which 47 were pregnant, with any number of implantations, and 11 were non-pregnant at E18.5. SCR and Slc38a2 were compared by ^1^Fisher’s exact test or ^2^Mann–Whitney U-test.

Placenta-specific *Slc38a2* knockdown reduced fetal weight by 11% compared with SCR controls (Figure 1C), consistent with our original hypothesis, and despite fewer Slc38a2KD fetuses per litter. Slc38a2KD also reduced placental weight by 18% (Figure 1D). When SCR and Slc38a2KD conceptuses were combined, fetal weight correlated with placental *Slc38a2* expression (Figure 1E), whereas placental weight did not (R = 0.305, *P*=0.052, *n*=41). The frequency distribution of fetal weights in Slc38a2KD conceptuses was left-shifted, compared with SCR conceptuses (Figure 1F) such that a greater proportion of Slc38a2KD fetuses (22%) were below the 10^th^ centile of control fetal weight (854 g, *P*=0.015, Fisher’s exact test), consistent with the FGR clinical phenotype.

To investigate the mechanism linking placenta-specific *Slc38a2* silencing with reduced placental weight, we also determined the activity of the nutrient-sensing amino acid response and mTOR signaling pathways in SCR and Slc38a2KD placentas, using Western blotting. Slc38a2KD affected neither abundance nor phosphorylation of eIF2α (Ser^51^), which is responsive to amino acid deprivation, or the read-outs of mTOR activity, S6 ribosomal protein (Ser^235/236^), eukaryotic translation initiation factor 4E-binding protein (4EBP1, Thr^37/46^) and protein kinase B (Akt, Ser^473^) (Supplemenatary Figure S4).

### Placenta-specific *Slc38a2* knockdown reduces placental System A amino acid transport

**Figure 2:**
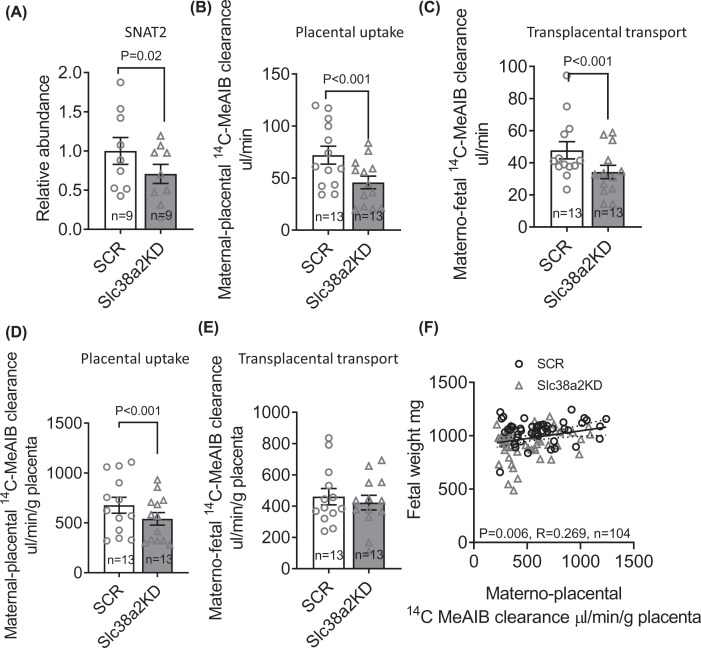
Placenta-specific *Slc38a2* knockdown reduces TPM SNAT2 abundance and placental System A amino acid transport (**A**) SNAT2 protein abundance in isolated TPMs from pooled SCR and Slc38a2KD placentas, determined by Western blot. Representative blot includes human placental microvillous membrane (MVM) sample, as positive control. (**B**–**E**) Placental uptake and transplacental transport of ^14^C-MeAIB in SCR (*n*=13 litters, representing 46 conceptuses) and Slc38a2KD conceptuses (*n*=13 litters, representing 58 conceptuses), expressed as absolute values (B,C) and per gram of placenta (D,E). Litter mean values for SCR and Slc38a2KD conceptuses compared by paired *t* test. *P*-values for significant differences (*P*<0.05) given in figure. Mean + SEM. (**F**) Correlation of fetal weight with placental ^14^C-MeAIB uptake for SCR (*n*=46) and Slc38a2KD (*n*=58) conceptuses. Points represent individual conceptuses. Pearson correlation coefficient (R) and *P*-value given in figure. Linear regression line with 95% confidence intervals shown.

**Figure 3:**
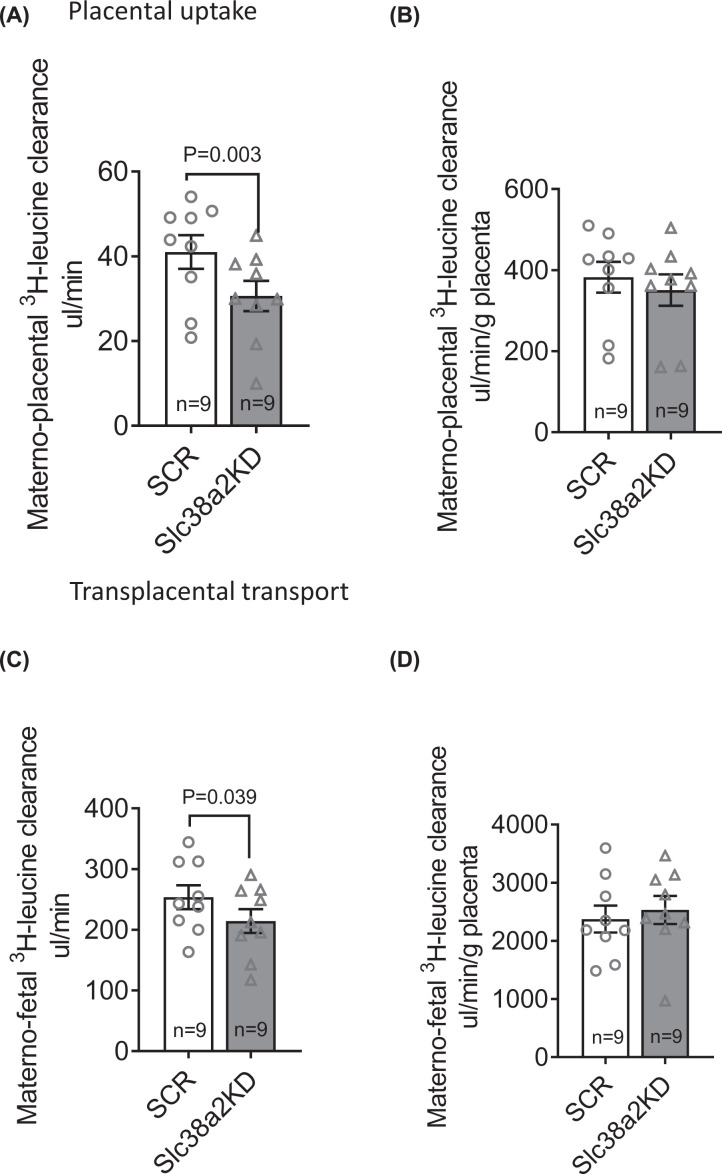
Placental System L amino acid transport in SCR and Slc38a2KD conceptuses (**A**,**B**) Placental uptake and (**C**,**D**) transplacental transport of ^3^H-leucine, expressed as absolute values (A,C) or per gram placenta (B,D). *n*=9 litters (33 SCR conceptuses, 29 Slc38a2KD conceptuses). Litter mean values for SCR and Slc38a2KD conceptuses compared by paired Student’s *t* test. *P*-values for significant differences (*P*<0.05) given in figure. Mean + SEM

Next, we used immunoblotting to determine SNAT2 protein abundance in the isolated TPM of E18.5 SCR and Slc38a2KD placentas, because membrane translocation of the transporter is required for cellular amino acid accumulation and known to be post-translationally regulated in trophoblasts [26]. TPM SNAT2 was 30% lower in Slc38a2KD than in SCR placentas, in line with *Slc38a2* mRNA expression (Figure 2A). Placental System A amino acid transport capacity was then determined by the *in vivo* unidirectional maternal–placental clearance (placental uptake) and maternal–fetal clearance (transplacental transport) of non-metabolizable ^14^C-MeAIB (see ‘Materials and methods’ section). Slc38a2KD reduced both placental uptake and transplacental transport of ^14^C-MeAIB, consistent with an overall reduction in placental System A transport capacity (Figure 2B,C). Placental uptake of ^14^C-MeAIB was also lower in Slc38a2KD than SCR conceptuses when expressed per gram of placental weight (Figure 2D). However, there was no significant difference between SCR and Slc38a2KD conceptuses when transplacental ^14^C-MeAIB transport was expressed per gram of placenta (Figure 2E), indicating that the mass-specific System A transport activity of the placenta was maintained, despite its smaller size. Fetal weight was positively correlated with placental uptake of ^14^C-MeAIB, when each conceptus within each litter was considered as an individual (Figure 2F). We similarly determined maternal–placental and maternal–fetal clearance of ^3^H-leucine, as read-outs of placental System L exchanger activity. Both placental uptake and transplacental clearance of ^3^H-leucine were lower in Slc38a2KD than SCR conceptuses as absolute values, but not when expressed per gram of placenta (Figure 3).

### Placenta-specific *Slc38a2* knockdown decreases fetal islet GSIS

**Figure 4:**
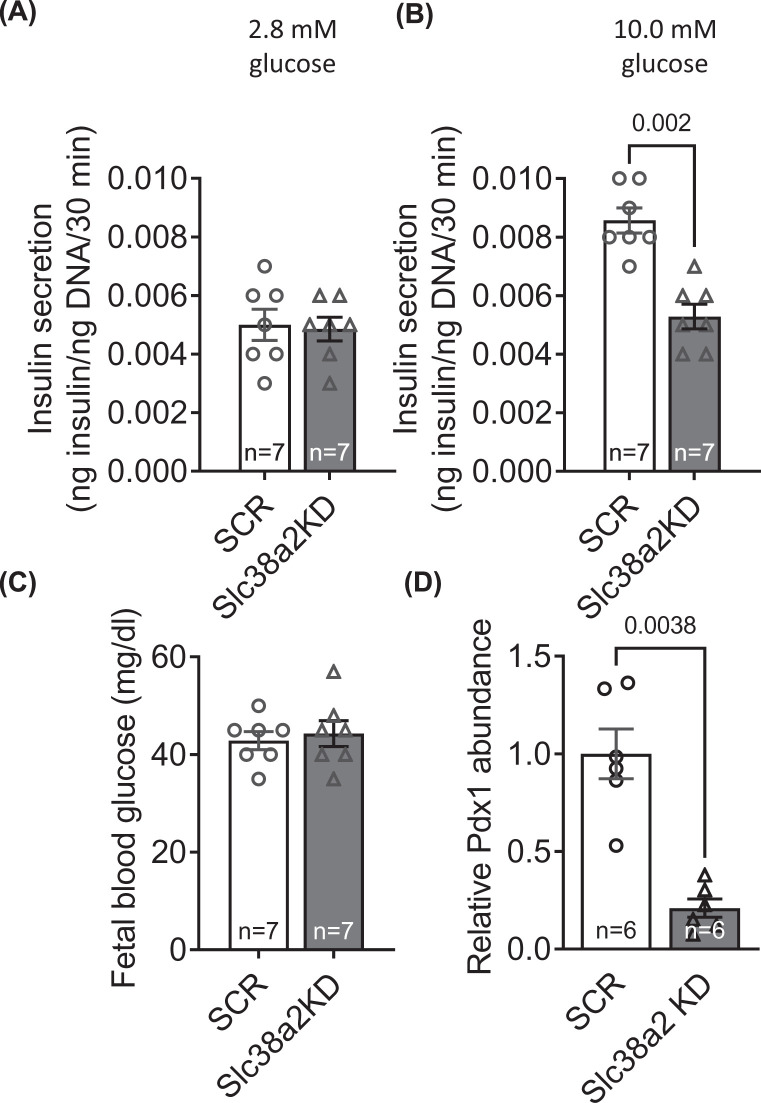
Placenta-specific Slc38a2 knockdown decreases fetal islet GSIS GSIS from mouse fetal islet cells. Pancreatic fetal islets were isolated, and GSIS determined in response to stimulation with (**A**) 2.8 mM and (**B**) 10.0 mM glucose for 60. (**C**) Fetal blood glucose was measured in trunk blood. *n*=7 litters/group. GSIS and blood glucose for SCR and Slc38a2KD conceptuses compared by paired Student’s *t* test. *P*-values for significant differences (*P*<0.05) given in the figure. Mean + SEM.

GSIS was measured *ex vivo* in pancreatic islets isolated from a subset of Slc38a2KD and SCR fetuses at E18.5. Islet GSIS did not differ between Slc38a2KD and SCR fetuses in the presence of 2.8 mM glucose but was significantly lower in Slc38a2KD fetuses than SCR fetuses at 10.0 mM glucose (Figure 4A,B). Fetal glucose concentrations were similar in the two groups (Figure 4C). Placenta-specific *Slc38a2* knockdown reduced fetal islet abundance of the transcription factor Pdx1, a master regulator of β cell differentiation (Figure 4D).

### Human placental *SLC38A2* gene expression is decreased in late- but not early-onset FGR

**Figure 5:**
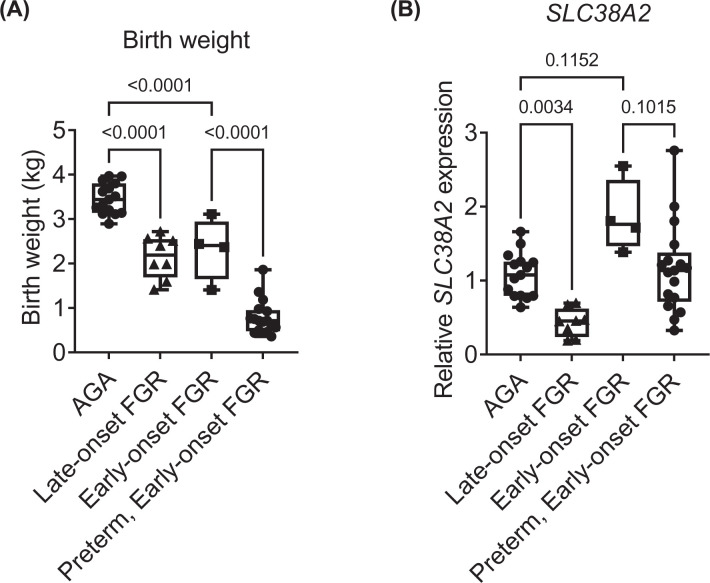
Human placental *SLC38A2* expression is decreased in late- but not early-onset FGR (**A**) Birth weights and (**B**) placental relative *SLC38A2* expression levels in AGA and FGR neonates, delivered at term (≥37 weeks gestation) or preterm (<37 weeks gestation, early-onset FGR only). Birth weights were statistically compared between groups by one-way ANOVA (*P*<0.001) with planned post-hoc comparisons using Sidak’s multiple comparisons test. *SLC38A2* expression was statistically compared between groups using Kruskal–Wallis test (*P*<0.001) with planned post-hoc comparisons using Dunn’s multiple comparisons test. *P*-values for post-hoc tests given in figure. AGA *n*=15, late-onset FGR *n*=8, term early-onset FGR *n*=4, preterm early-onset FGR *n*=17. Boxes are median with IQR, whiskers are range.

Finally, we quantified *SLC38A2* expression in human placentas collected at term from AGA, early- and late-onset FGR neonates, with informed consent in a U.K. hospital setting. Because early-onset FGR is frequently associated with preterm delivery, we separately compared *SLC38A2* expression in early-onset FGR placentas delivered at term with those delivered preterm (<37 weeks of gestation). Maternal age and pre-pregnancy body mass index (BMI) and the proportional distributions of fetal sex and delivery mode were similar in AGA and FGR groups, irrespective of gestational age at FGR onset and at delivery (Table 2). Maternal ethnicity differed between study groups, with the late-onset FGR group containing the largest proportion of Asian, Black or Mixed ethnicity subjects and the AGA group containing the smallest proportion of subjects of these ethnicities. Women with FGR fetuses were more likely than women with AGA fetuses to be diagnosed with a hypertensive disorder of pregnancy, defined as pregnancy-induced hypertension, pre-eclampsia or HELLP syndrome. Hypertensive disorder of pregnancy was highest in the preterm, early-onset FGR group. As expected, women in the preterm, early-onset FGR group delivered earlier than the other three groups, by ∼10 weeks on average. Placental weight differed significantly between AGA and FGR groups but was not recorded for all subjects (Table 2). Both birth weight and placental *SLC38A2* expression were lower in late-onset FGR pregnancies than AGA pregnancies, by 38 and 60%, respectively (Figure 5). Contrastingly, early-onset FGR pregnancies delivered at term had birth 1weights that were 33% lighter than AGA control birthweights, but their placental *SLC38A2* expression levels did not differ significantly, and tended to be higher than control values (Figure 5B). Placental *SLC38A2* expression also tended to be higher in term-delivered early-onset FGR pregnancies compared with their preterm-delivered counterparts, which had even lower birth weights (Figure 5A,B).

**Table 2:** Demographic characteristics of human AGA and FGR study participants

	Term delivery			Preterm delivery	*P*-value
	AGA	Late-onset FGR	Early-onset FGR	Early-onset FGR	
	*n*=15	*n*=8	*n*=4	*n*=17	
**Maternal age (years)**	38.0 (31.0–39.0)	32.5 (31.0–34.8)	32.0 (25.3–39.5)	35.0 (31.0–39.5)	0.459^1^
**Maternal BMI (kg/m^2^)**	23.0 (22.0–28.0)	22.0 (22.0–31.0)	23.0 (21.5–32.0)	24.0 (23.0–28.0)	0.445^2^
**Ethnicity (number Asian, Black or Mixed)**	2 (13%)	6 (75%)	1 (25%)	5 (29%)	0.025^3^
**Hypertensive disorder of pregnancy (number with any diagnosis)**	0 (0%)	1 (13%)	1 (25%)	7 (41%)	0.034^3^
**Fetal sex (number male)**	10 (67%)	4 (50%)	1 (25%)	6 (35%)	0.256^3^
**Mode of delivery (number vaginal)**	4 (27%)	1 (13%)	2 (50%)	1 (6%)	0.148^3^
**Gestational age (weeks)**	39.1 (39.0–39.6)	37.5 (36.9–38.5	38.4 (37.6–40.5)	28.0^5^ (27.1–31.0)	<0.001^2^
**Placental weight (g)^6^**	491 (428–521)	351^4^ (240–365)	584	142^5^ (135–185)	<0.001^1^

Continuous variables are given as median (interquartile range) and compared by one-way ANOVA^1^ with Sidak’s multiple comparison *post-hoc* or by Kruskal–Wallis test^2^ with Dunn’s multiple comparison *post-hoc.* ^4^Significantly different from AGA, ^5^significantly different from early-onset FGR delivered at term. Categorical variables are given as number (% of *n*) and compared by chi-squared test^3^. ^6^Placental weights were collected for a subset of study participants: AGA *n*=9, late-onset FGR *n*=6, term early-onset FGR *n*=1, preterm early-onset FGR *n*=5.

Within subjects delivering at term, placental *SLC38A2* expression correlated with placental weight (*P*=0.005, R = 0.664, *n*=16) and gestational age at delivery (*P*=0.008, R = 0.501, *n*=27) but did not correlate with any of the other continuous independent variables (Supplementary Table S2). When FGR status and potential confounding variables (gestational age, maternal ethnicity and hypertensive disorder) were entered into a multiple linear regression model to predict placental *SLC38A2* expression at term, the overall effect of FGR status remained significant (main effect: *P*<0.001; regression model: *P*<0.001, R^2^ = 0.769, degrees of freedom = 21). Regression coefficients for the adjusted effect of each type of FGR, compared with AGA, further indicated decreased *SLC38A2* expression in late-onset FGR (*P*=0.058, β= −0.389) and increased placental *SLC38A2* expression in early-onset FGR (*P*<0.001, β = 0.869) (Supplementary Table S3).

## Discussion

The present study shows, for the first time, that placenta-specific knockdown of the System A amino acid transporter *Slc38a2*/SNAT2 causes FGR in mice, consistent with our original hypothesis. Lower fetal weight in Slc38a2KD transduced conceptuses was accompanied by diminished fetal viability, lower placental weight, lower maternal–fetal MeAIB clearance and lower fetal islet GSIS near term, compared with control transduced conceptuses. The study also provides the first demonstration that human placental *SLC38A2* expression is reduced at term in FGR pregnancies compared with AGA pregnancies, specifically when FGR is late in onset, occurring after 32 weeks of gestation, but not when it is diagnosed before. Our study therefore proves that normal trophoblast *Slc38a2*/SNAT2 expression and function are necessary for normal fetal growth and supports the concept that impaired placental System A amino acid transport mechanistically contributes to human late-onset FGR.

The 11% reduction in fetal weight with placenta-specific *Slc38a2* knockdown was similar in magnitude to that observed in mouse embryos with global *Slc38a2* deletion [32] and in rat embryos following inhibition of System A amino acid transport using unlabeled MeAIB infusion [31]. These findings are all consistent with the physiological role of SNAT2 as an active transporter of amino acids across the rodent placenta, where it is abundantly expressed in the labyrinthine TPM and correlates with fetal size [28,63,64]. FGR in our study was attributable to a deficiency of trophoblast membrane SNAT2 and overall functional reduction in System A-mediated amino acid delivery to the umbilical circulation, because transplacental MeAIB clearance was 23% lower in Slc38a2KD than SCR conceptuses. However, *Slc38a2* silencing also caused placental growth restriction, which preceded FGR, alongside diminished placental System A amino acid uptake per unit mass of tissue. The fact that *transplacental* MeAIB clearance per gram of placenta was not changed therefore suggests that the fetal effect of *Slc38a2* knockdown was partly mediated by smaller placental size, and consequently reduced exchange surface area, as well as lower SNAT2 transporter density in the TPM. Lower placental weight in Slc38a2KD conceptuses was not related to inhibition of the nutrient-sensing amino acid response and mTOR signaling pathways, which regulate trophoblast growth *in vitro* [65,66]. Instead, SNAT2 deficiency in the TPM probably directly reduced intracellular amino acid availability for cellular accretion or proliferation. Indeed, mouse placentas genetically lacking *Slc38a4/*SNAT4 exhibit mid-gestation hypoplasia, with reduced expression of cell cycle-related genes and lower numbers of proliferating trophoblasts in the chorionic plate and labyrinthine zone [32]. In contrast with our findings, global genetic deletion of *Slc38a2* does not alter placental weight at term [32]. This may suggest that compensatory processes maintain placental size during differentiation of the trophectoderm when *Slc38a2* is deleted by zygotic CRISPR-*Cas9* injection, albeit the number of *Slc38a2-*deficient placentas analysed in the previous study was very small.

Near-term fetal viability rate was reduced by 33% in Slc38a2KD embryos, whereas implantation rates were similar to controls, in line with the effect of global *Slc38a2* deletion [32,33]. Therefore, a greater proportion of Slc38a2KD embryos died after implantation but before term. This finding reflects the increased incidence of stillbirth and perinatal death seen in FGR human fetuses [67–69] and supports the importance of placental SNAT2 for normal fetal development. It indicates that SNAT2 may be involved in post-implantation development, earlier in gestation. Certainly, SNAT2 is abundantly expressed in the peri-implantation mouse embryo and plays a role in embryonic stem cell differentiation, although its role in extraembryonic cells in early gestation is not well established [70,71].

The effects of placenta-specific *Slc38a2* knockdown on fetal growth were generally milder than those of glucocorticoid excess, hypoxia, protein restriction or nitric oxide deficiency in pregnant rodents, which are similarly associated with reduced System A amino acid transport [28,34–36,38]. This is probably explained by the tendency for these perturbations to reduce fetal delivery of other nutrients and oxygen by altering uteroplacental blood flow. Additionally, some of these insults are associated with placental down-regulation of the other System A amino acid transporters, *Slc38a1/*SNAT1 or *Slc38a4/*SNAT4 [29,34,38], which may have additive effects on net transplacental amino acid transport. Placental *Slc38a1* and *Slc38a4* expression was maintained at normal levels in our study, by design. The relative importance of *Slc38a2/*SNAT2 compared with the other two placental SNAT transporter isoforms is still being established and appears to vary with species. Global deletion of either *Slc38a1, Slc38a2* or *Slc38a4* reduces fetal growth in mice [32], while studies in isolated microvillous membrane indicate that SNAT1 and SNAT2 mainly mediate neutral amino acid uptake in rat syncytiotrophoblast [72]. In humans, *in vitro* experiments demonstrate that SNAT1, SNAT2 and SNAT4 all contribute to trophoblast System A-mediated amino acid uptake, depending on gestational age [23,24,72,73], but placental SNAT2 abundance is most closely related to term fetal growth [22,26]. The present study shows that exclusively reducing placental *Slc38a2* expression is sufficient to impair maternal–fetal System A amino acid transport and fetal growth. Notably, FGR occurred even though placental *Slc38a2* silencing did not affect net placental uptake or transplacental transport of the essential amino acid, leucine. This finding was unexpected, given that leucine transport is mediated by System L transporters [19], the activity of which is believed to depend on the intracellular concentration of non-essential amino acids accumulated by System A [20]. This observation may be partly explained by computational modeling data suggesting that placental L-type amino acid transporters are not obligate exchangers but can also act as facilitated transporters [74].

Although reduced fetal weight in our study is partly explained by low amino acid availability for tissue accretion, it may also be indirectly attributed to fetal hyperinsulinaemia, due to impaired pancreatic islet function. Our finding of reduced GSIS in Slc38a2KD fetal pancreatic islets reflects that in growth restricted human and sheep fetuses with reduced umbilical amino acid concentration, β-cell mass and pancreatic expression of the transcription factor *Pdx1* [75–79]. *Pdx1* is a key differentiation initiator in pancreatic progenitor cells and activator of β-cell specific genes, and is regulated by amino acids, including the System A substrate, glutamine [80]. Therefore, at a cellular and molecular level, reduced GSIS in pancreatic islets from Slc38a2KD fetuses is most likely due to deficient β-cell development caused by amino acid deprivation and *Pdx1* down-regulation.

The physiological effects of placental *Slc38a2* silencing were not in proportion to the degree of knockdown of mRNA expression, which was decreased by more than 50%. This reflects the limitation of using RNAi methodology to study placental membrane-bound transporter function, which is also governed by the rates of translation and, particularly, trafficking of the transporter proteins to the plasma membrane [27]. Furthermore, lower viability in Slc38a2KD fetuses may have contributed to an underestimation of the effect on nutrient transport and fetal growth, because fetal weight in rodents is inversely related to the number of fetuses in each horn [81]. Although the experimental design controlled for effects of overall litter size, by transferring control and Slc38a32KD embryos to contralateral horns of each recipient uterus, the relative difference in fetal weight and amino acid transport would most likely have been larger if we controlled the number of viable fetuses per horn. The full magnitude of the effect of placenta-specific *Slc38a2* silencing may also have been masked if those embryos with greatest functional knockdown of SNAT2 were resorbed prior to the E18.5 analyses. Nevertheless, our study demonstrates that lentiviral shRNA-mediated gene silencing is effective as a tool to study placental nutrient transporter function in mice.

This is the first study to determine human placental *SLC38A2* expression in well-defined early- and late-onset FGR subjects delivering at term. The reduction in *SLC38A2* gene expression in placentas with late-onset FGR was consistent with, but greater in magnitude than, that reported in another cohort, which had a mean gestational age of ∼33 weeks and most likely represented a mixed population of early- and late-onset FGR pregnancies [26]. The data therefore strongly support a mechanistic role for placental *SLC38A2* deficiency in late-onset FGR. By contrast, the lack of reduction in *SLC38A2* expression in early-onset FGR placentas was unexpected. It may suggest that amino acid transport is causatively less important than factors such as impaired spiral artery remodeling and uteroplacental blood flow in early-onset FGR [44]. Alternatively, early-onset FGR may be underpinned by reduced post-translational trafficking of SNAT2 to the TPM, due to suppressed mTOR signaling [22], rather than decreased *SLC38A2* transcription *per se.* We were unable to measure membrane localized transporter abundance or System A activity using the small amounts of human placental material collected in this study. The analyses of human placentas were also limited by the small number of patients with early onset FGR that reached term for delivery; the lack of preterm AGA control placentas; and our inability to control for possible effects of maternal ethnicity or hypertensive disorders, which differed between AGA and FGR subjects. Nevertheless, the data provide compelling evidence that early- and late-onset FGR are underpinned by different mechanisms, with reduced placental amino acid transport critical in the latter.

Overall, the present study is the first demonstration of a cause-and-effect relationship between placental *Slc38a2*/SNAT2 expression and fetal growth. Prior to the present study, there was no definitive evidence that SNAT2 deficiency mechanistically contributes to FGR. Given that System A amino acid transporter activity is compromised more severely in human FGR placentas (>70% [22]) than in this study (23%), the results strongly indicate that diminished fetal amino acid supply is an underlying cause. Currently, there is no specific treatment of FGR in the clinic. Our findings suggest that interventions that augment placental System A amino acid transport capacity may improve fetal growth and outcomes in severe FGR. These interventions may be most effective in women diagnosed with late-onset FGR, which constitutes the overwhelming majority of FGR cases.

## Clinical perspectives

- FGR is an obstetric disease that severely compromises perinatal survival and long-term health of the infant. The biological mechanisms underlying FGR are unknown, preventing us from developing treatments.
- We show that experimental knockdown of *Slc38a2* in the mouse placenta reduces fetal weight at the end of gestation, demonstrating a cause-and-effect relationship between low placental Slc38a2/SNAT2 expression and FGR *in vivo*. We also show that human placental Slc38a2 expression is specifically reduced in late-onset FGR.
- Our findings support the concept that placental SNAT2 deficiency underpins human FGR and suggest that interventions that augment placental amino acid transport could improve fetal growth and perinatal outcomes in severe FGR.

## Supplementary Material

Supplementary Figures S1-S5 and Tables S1-S4

## Data Availability

The authors confirm that the data supporting the findings of the present study are available within the article, its supplementary materials and from the corresponding author [Owen R. Vaughan] upon reasonable request.

## Open Access

Open access for this article was enabled by the participation of University College London in an all-inclusive *Read & Publish* pilot with Portland Press and the Biochemical Society under a transformative agreement with JISC.

## Abbreviations

AGA: appropriate for gestational age
EFW: estimated fetal weight
EGFP: enhanced green fluorescent protein
eIF2: eukaryotic initiation factor 2
FGR: fetal growth restriction
GFP: green fluorescent protein
GSIS: glucose-stimulated insulin secretion
HELLP: haemolysis, elevated liver enzymes and low platelet count
MeAIB: methylaminoisobutyric acid
mTOR: mechanistic target of rapamycin
Pdx1: pancreatic and duodenal homobox 1
SCR: scrambled
shRNA: small hairpin RNA
SNAT: sodium-coupled neutral amino acid transporter
TPM: trophoblast plasma membrane
4EBP1: eukaryotic translation initiation factor 4E-binding protein
