# Supplementary material for: Placenta-specific Slc38a2/SNAT2 knockdown causes fetal growth restriction in mice
Source: Clin Sci (Lond). 2021 Aug 31;135(17):2049–66. doi: 10.1042/CS20210575 (PMC8410983; doi:10.1042/CS20210575)
Supplement: Supplementary Figures S1-S5 and Tables S1-S4 [file CS-2021-0575_supp.pdf]

SUPPLEMENTAL TABLES AND FIGURES

Placenta-specific *Slc38a2*/SNAT2 knockdown causes fetal growth restriction in mice.

Owen R. Vaughan[1,2]*, Katarzyna Maksym[2], Elena Silva[1], Kenneth Barentsen[1], Russel V. Anthony[3], Thomas L. Brown[4], Sara L. Hillman[2], Rebecca Spencer[2,5], Anna L. David[2], Fredrick J. Rosario[1], Theresa L. Powell[1,6] and Thomas Jansson[1]

[1]Department of Obstetrics and Gynecology, University of Colorado Anschutz Medical Campus, Aurora, CO, USA, 80045
[2]Department of Maternal and Fetal Medicine, Elizabeth Garrett Anderson Institute for Women's Health, University College London, UK, WC1E 6HX
[3]Department of Biomedical Sciences, Animal Reproduction and Biotechnology Laboratory, Colorado State University, Fort Collins, CO, USA, 80523
[4]Department of Neuroscience, Cell Biology and Physiology, Wright State University Boonshoft School of Medicine, Dayton, OH, USA, 45435
[5]Leeds Institute of Cardiovascular and Metabolic Medicine, University of Leeds, UK, LS2 9NL
[6]Department of Pediatrics, University of Colorado Anschutz Medical Campus, Aurora, CO, USA, 80045
*Corresponding and lead author

Contact information:
Owen R. Vaughan
EGA Institute for Women's Health,
86-96 Chenies Mews,
London, WC1E 6HX
United Kingdom
owen.vaughan@CUAnschutz.edu
ORCID ID: https://orcid.org/0000-0001-7537-3264
Twitter: @OwenRVaughan

Conflict of interest statement.

The authors have declared that no conflict of interest exists.

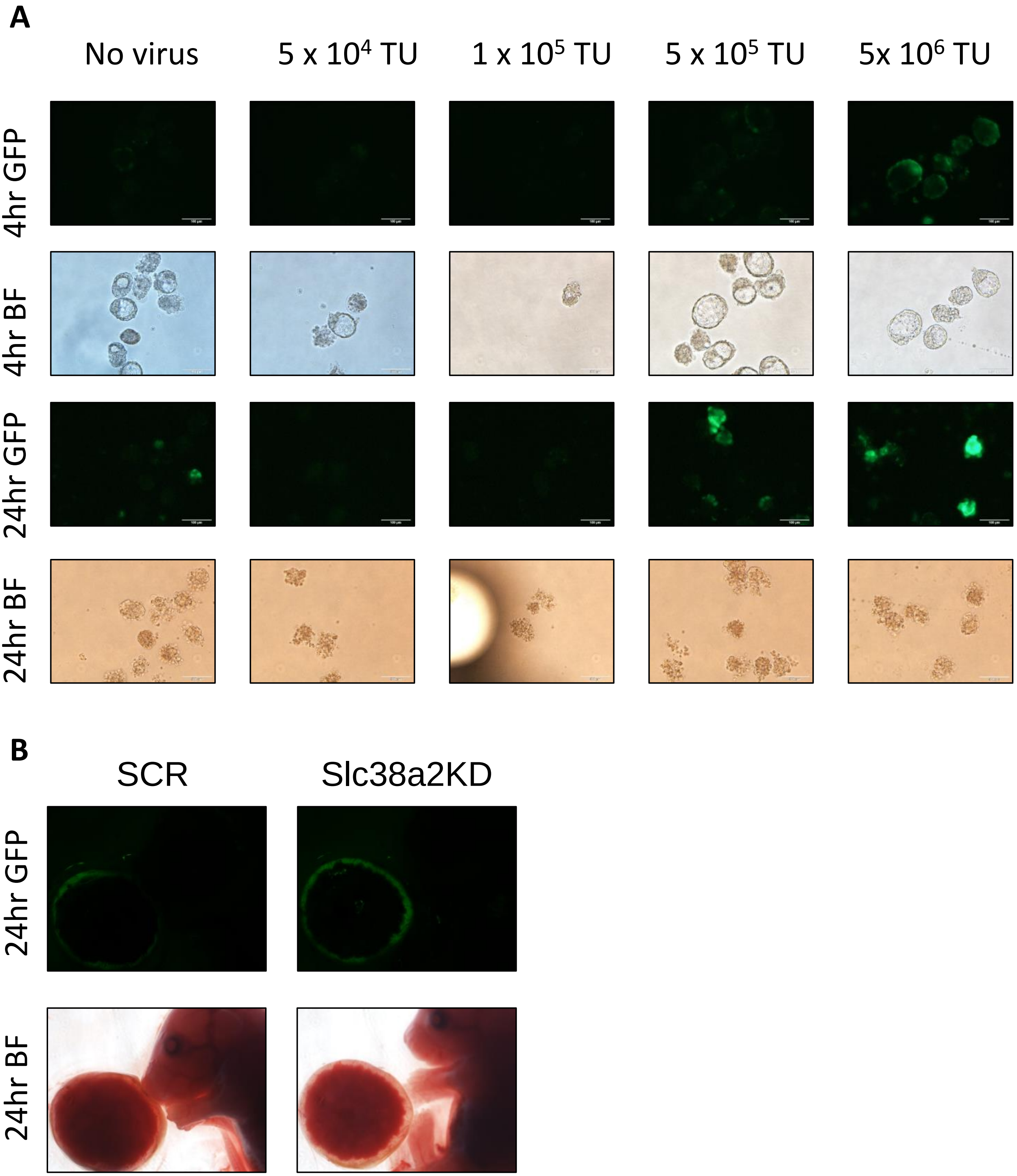

Fig. S1 GFP reporter expression in lentivirus transduced embryos. (A) Fluorescence and bright field (BF) images of blastocysts transduced with varying titers (TU, transforming units) of Slc38a2KD lentivirus, immediately after 4 hr transduction (E3.5) or 24 later (E4.5). (B) Fluorescent and bright field images of embryos collected near term (E17.5) following transduction with SCR or Slc38a2KD lentivirus.

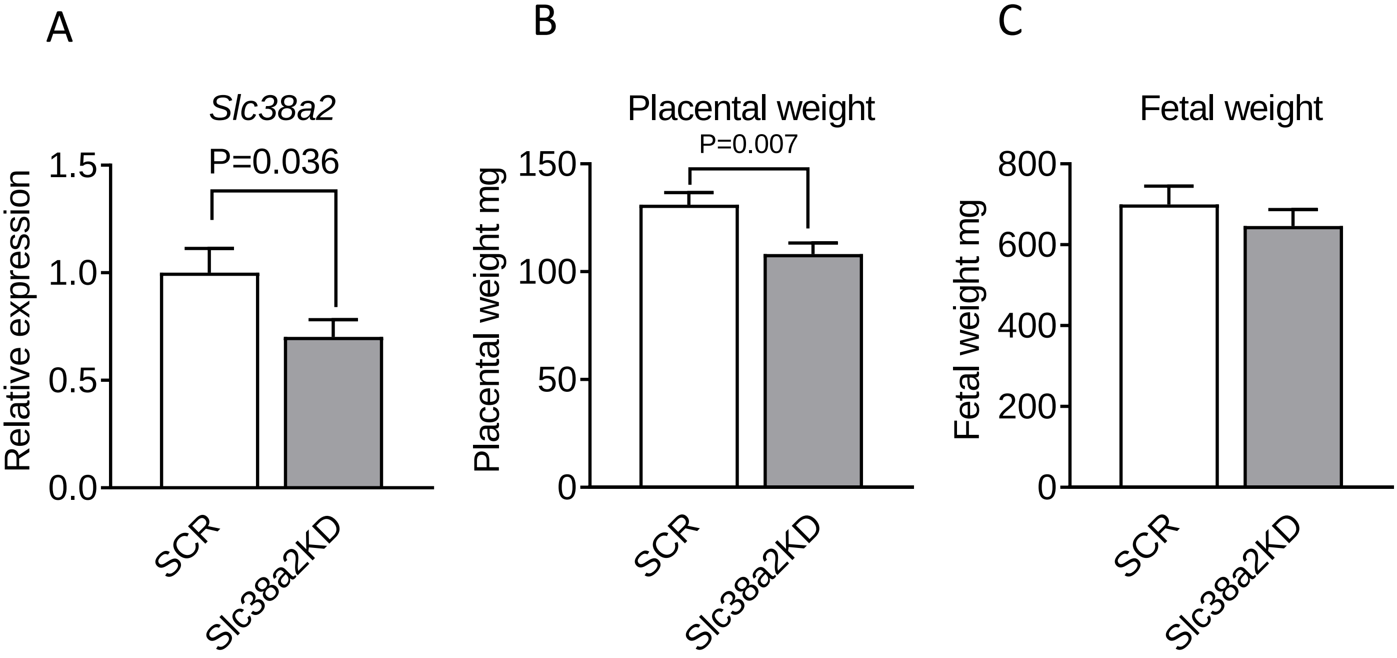

Fig. S2 Slc38a2 gene expression (A), placental (B) and fetal weights (C) in SCR and Slc38a2 transduced embryos at E17.5. SCR, n=11 conceptuses and Slc38a2, n=14 conceptuses from four litters, each with at least 1 conceptus transduced with each virus. Mean + SEM. P values for statistically significant differences (P<0.05 by Student's t-test) between SCR and Slc38a2 given in figure.

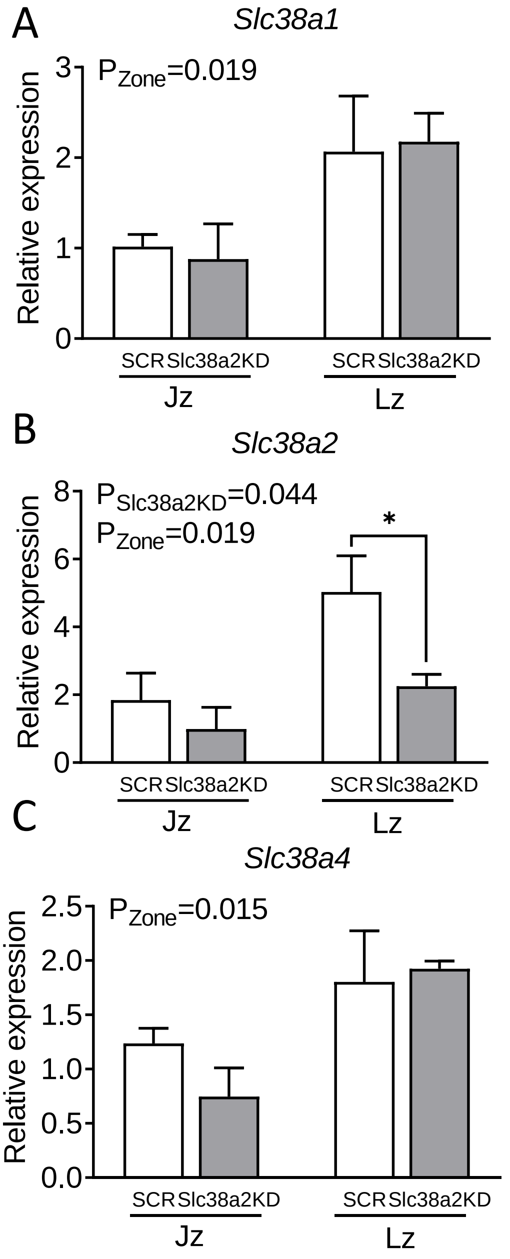

Fig. S3 ***Slc38a1*** **(A),** ***Slc38a2*** **(B) and** ***Slc38a4*** **(C) gene expression in dissected junctional (Jz) and labyrinthine (Lz) zones of SCR and Slc38a2KD placentae.** n=3 litters (15 SCR placentas, 9 Slc38a2KD placentas). Main effects of Slc38a2KD ($P_{Slc38a2KD}$) and zone (P_{zone}) determined by two-way ANOVA. P values for significant overall effects ($P<0.05$) given in figure. *, $P<0.05$ Fisher's LSD post-hoc test. Mean + SEM

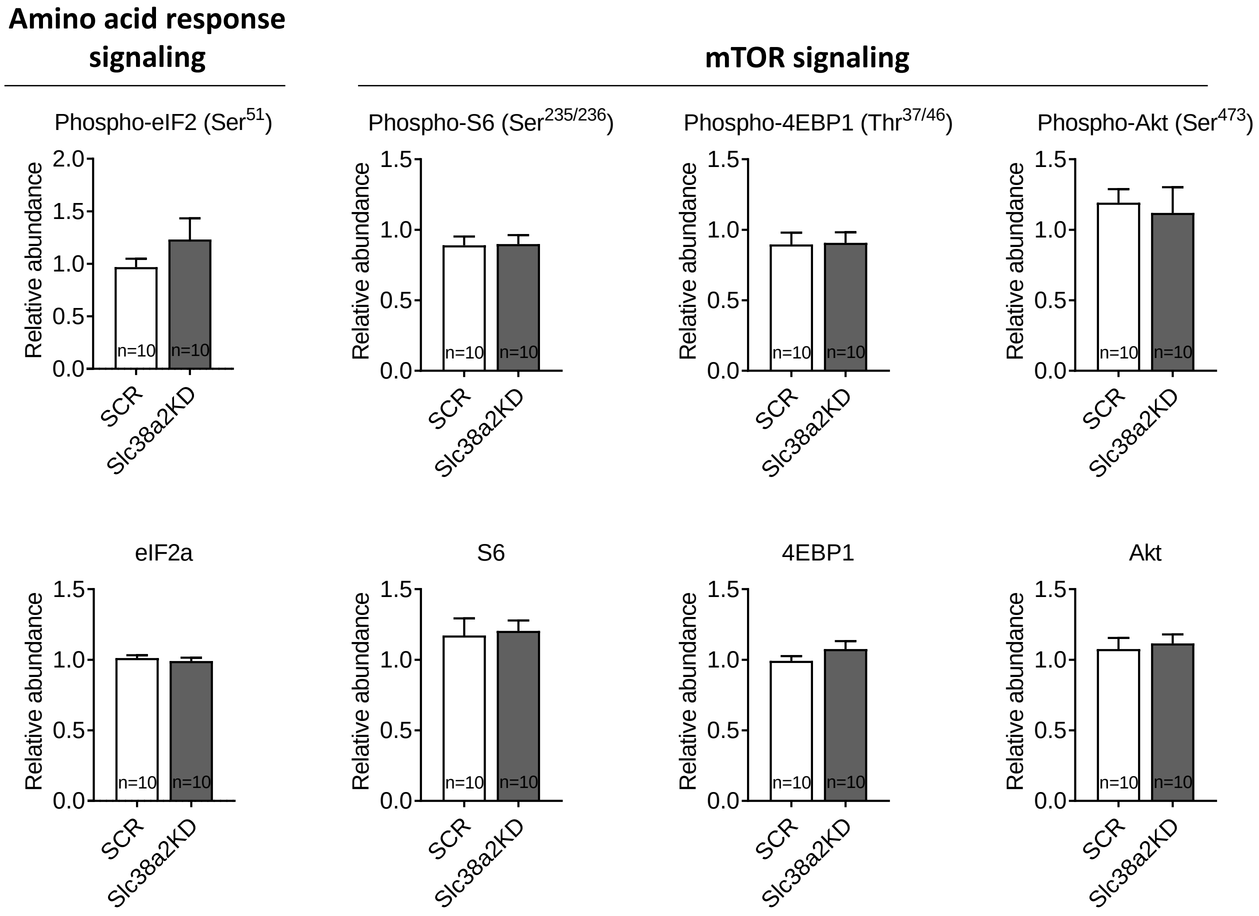

Fig. S4 Amino acid response and mTOR signalling pathway activity in SCR and Slc38a2KD placentae. Phosphorylated and total protein abundance determined by western blot in pooled placental homogenates. SCR and Slc38a2KD pools compared by paired Student's t –test. No significant differences at $P<0.05$. Mean + SEM

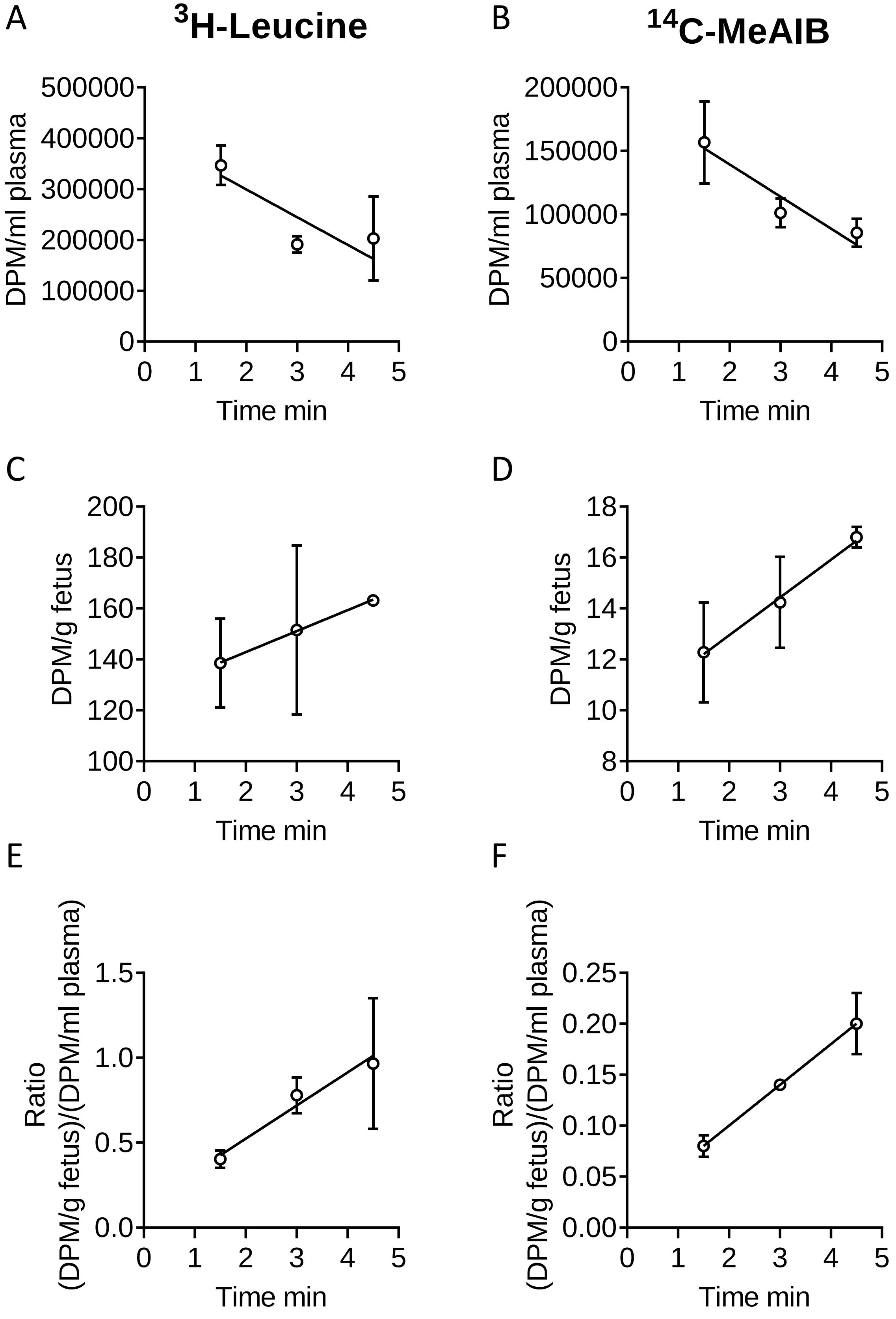

Fig. S5 Time courses of maternal and fetal radioactivity up to 4.5 min after bolus infusion of ^{14}C-MeAIB and ^{3}H-leucine. ^{3}H (A, C, E) and ^{14}C radioactivity (B, D, F), in decays per minute (DPM) determined by liquid scintillation counting. (A, B) Maternal plasma radioactivity per ml, (C, D) fetal radioactivity per gram bodyweight, (E, F) ratio of maternal and fetal radioactivity. Points are mean ± SEM. 1.5 min, n=4; 3 min, n=3; 4.5 min, n=2. Dependence of radioactivity on time from infusion determined by least squares linear regression .(A-D) Slope does not differ significantly from zero ($P>0.05$). (E) $P=0.03$, $R^2=0.53$, $y=(0.20 \pm 0.07)x + (0.13 \pm 0.20)$. (F) $P<0.01$, $R^2=0.86$, $y=(0.04 \pm 0.01)x + (0.02 \pm 0.02)$.

Table S1 Implantation and viability rates for SCR and Slc38a2KD transduced embryos at E17.5

	SCR	Slc38a2KD	P value
**Total embryos transferred (n)**	49	53	
**Implanted (n)**	11	17	
**%**	22%	32%	0.375[a]
**Viable fetus at E18.5**	11	17	
**%**	22%	32%	0.375[a]
**Number of fetuses per uterine horn (median, min-max)**	3 (1-4)	3 (1-7)	0.971[b]

Embryos were transferred to 6 recipient dams, of which 5 were pregnant, with any number of implantations, and 1 was non-pregnant on E17.5. SCR and Slc38a2 were compared by [a]Fisher's exact test or [b]Mann-Whitney U-test.

Table S2 Relationship between human placental *SLC38A2* and clinical characteristics of AGA and FGR subjects delivered at term.

		Placental *SLC38A2*	Maternal age	Maternal BMI	Gestational age	Placental weight	Birth weight
**Placental *SLC38A2***	**r**	**1.000**	**0.120**	**-0.050**	**0.501**	**0.664**	**0.256**
	Confidence interval		-0.272 to 0.478	-0.429 to 0.344	0.150 to 0.740	0.251 to 0.873	-0.137 to 0.580
	P		*0.550*	*0.810*	*0.008*	*0.005*	*0.198*
	n	27	27	26	27	16	27
**Maternal age**	**r**		**1.000**	**0.144**	**0.195**	**0.215**	**0.343**
	Confidence interval			-0.258 to 0.503	-0.200 to 0.535	-0.314 to 0.642	-0.0426 to 0.640
	P			*0.484*	*0.330*	*0.424*	*0.080*
	n		27	26	27	16	27
**Maternal BMI**	**r**			**1.000**	**-0.098**	**-0.071**	**-0.001**
	Confidence interval				-0.468 to 0.301	-0.563 to 0.458	-0.388 to 0.386
	P				*0.634*	*0.800*	*0.996*
	n			26	26	15	26
**Gestational age**	**r**				**1.000**	**0.806**	**0.709**
	Confidence interval					0.516 to 0.930	0.451 to 0.858
	P					*<0.001*	*<0.001*
	n					16	27
**Placental weight**	**r**						**0.877**
	Confidence interval						0.674 to 0.957
	P						*<0.001*
	n						16
**Birthweight**	**r**						**1.000**
	Confidence interval						
	P						
	n						27

Interdependence of variables determined by Pearson's correlation.

Table S3 Estimated parameters for multiple linear regression mode to predict effect of FGR status on term placental *SLC38A2* expression, adjusted for confounding variables.

Variable	β	95% Confidence interval	P value
**Intercept**	-4.349	-9.87 to 1.17	0.116
**Late-onset FGR**	-0.389	-0.793 to 0.0150	0.058
**Early-onset FGR**	0.869	0.503 to 1.23	<0.001
**Ethnicity (Asian, black or mixed)**	-0.072	-0.413 to 0.270	0.667
**Gestational age**	0.139	-0.00232 to 0.279	0.054
**Hypertension in pregnancy**	-0.044	-0.584 to 0.496	0.866

Regression model: Placental *SLC38A2* ~ Intercept + FGR + Ethnicity + Gestational age + Hypertension in pregnancy. Reference levels for FGR status, ethnicity and hypertension were AGA, white and no hypertension, respectively. n=27 subjects

Table S4 Primer sequences used for qRT-PCR in mouse tissues.

Target mRNA	Forward primer	Reverse Primer	Reference
Slc38a1	CTTCTCCAGATTTCGTGCCG	AGGGAGAGAGAGAGAAGCCA	(Matoba et al., 2019)
Slc38a2	ACATAAGGCGTATGGTCTG	TACCACAACCCATTTGTATC	(Uno et al., 2015)
Slc38a4	GGCTTCTTCTGCCACTATGC	AAGACCAAAGCCCCAATCTT	(Matoba et al., 2019)
RNA28S	AAGTCCTTCTGACTGAGGCC	ATTCCCAAGCAACCCGACTC	(Uno et al., 2015)
